# Factors associated with violence in bipolar disorder versus major depressive disorder: insights from a retrospective cross-sectional study of 141 aged patients with mood disorders

**DOI:** 10.3389/fpsyt.2025.1700530

**Published:** 2026-01-12

**Authors:** Junrong Ye, Tingwei Zhou, Lingli Lei, Lili Mo, Jiao Chen, Wen Wang, Yanheng Wei, Xueling Lu, Lexin Yuan, Shengwei Wu, Zezhi Li, Dandan Zheng, Aixiang Xiao

**Affiliations:** 1 The Affiliated Brain Hospital, Guangzhou Medical University, Guangzhou, China; 2 School of Nursing, Guangzhou Medical University, Guangzhou, China; 3 The Affiliated Brain Hospital, Guangzhou Medical University, Key Laboratory of Neurogenetics and Channelopathies of Guangdong Province and the Ministry of Education of China, Guangzhou Medical University, Guangzhou, China; 4 Department of Nursing, Dongguan Mental Health Center, Dongguan, China; 5 Department of Nursing, Nanning Fifth People’s Hospital, Nanning, China

**Keywords:** cross-sectional study, elderly, factors, mood disorders, violence

## Abstract

**Objective:**

This study aims to explore the risk factors and protective factors associated with violence in patients with mood disorders and to identify the differences in these risk factors between clinical subtypes: bipolar disorder (BD) and major depressive disorder (MDD).

**Methods:**

A retrospective cross-sectional investigation was conducted from February 2021 to March 2024. Participants were consecutively sampled from the geriatric psychiatric ward of a tertiary psychiatric hospital. The general information questionnaire, Brøset Violence Checklist (BVC), Geriatric Depression Scale (GDS), and Functional Activity Questionnaire (FAQ) were used to evaluate the risk of violence, depressive syndromes, and activities of daily living of the participants.

**Results:**

Among the 141 elderly patients with mood disorders, 40 (28.4%) were diagnosed with bipolar disorder and 101 (71.6%) with major depressive disorder. The sample included 34.8% males and 65.2% females, with an average age of 67.42 ± 6.91 years. Regarding educational attainment, 12.1% had completed a bachelor’s degree or higher, 22.7% had completed senior school or an associate degree, and the majority (65.2%) had received education at the junior high school level or below. Unhealthy lifestyles, such as smoking and alcohol use, were relatively uncommon, with 7.1% smoking and 5% consuming alcohol. Univariate analysis of elderly patients with mood disorders revealed that gender, smoking status, first admission, type of admission, length of hospitalization, and restraint condition were significantly related to the risk of violence (*P* < 0.05). Additionally, the GDS and FAQ scores showed statistically significant differences (*P* < 0.05). Multiple linear regression analysis indicated that abnormal GDS scores (*β* = -0.424, *P* < 0.001), restraint condition (Yes) (*β* = 0.181, *P* < 0.05), and length of hospitalization (>14 days) (*β* = 0.145, *P* < 0.05) were significant factors influencing the risk of violence in hospitalized elderly patients with mood disorders. Specifically, for patients with MDD, abnormal GDS scores (*β* = -0.207, *P* < 0.05) and restraint condition (Yes) (*β* = 0.437, *P* < 0.001) were significant factors. For patients with BD, the length of hospitalization (>14 days) (*β* = 0.260, *P* < 0.05) was a significant factor influencing the risk of violence.

**Conclusion:**

Patients with mood disorders have a potential risk of violence, and the clinical factors influencing this risk differ between clinical subtypes. Therefore, given these distinct risk profiles across subtypes, precise risk assessment and early intervention are necessary to mitigate the risk of violence in these patients.

## Introduction

Mood disorders are a category of neuropsychiatric illnesses primarily characterized by mood disturbances. Individuals with mood disorders experience single or recurrent episodes of mood changes, such as severe depression and mania (1). According to the Diagnostic and Statistical Manual of Mental Disorders (DSM-V), mood disorders are divided into two groups: bipolar and related disorders and major depressive disorder (2). Mood disorders are among the most common and disabling mental health conditions worldwide (3). The global population is aging rapidly, with the number of people over 60 expected to increase from 605 million to 2 billion (4). Approximately 25% of individuals with bipolar disorder (BD) and 5% of those with major depressive disorder (MDD) are 60 years or older (5, 6). This proportion will likely grow in tandem with global demographic changes (6).

The World Health Organization (WHO) defines violence as: “the intentional use of physical force or power, threatened or actual, against oneself, another person, or against a group or community, that either results in or has a high likelihood of resulting in injury, death, psychological harm, maldevelopment, or deprivation” (7). Elderly individuals with mood disorders are often considered vulnerable to violence due to physical decline, interpersonal conflicts and disorientations (8, 9). However, older adults with mood disorders may also be more prone to engaging in aggressive behaviors (10). Notably, individuals with mood disorders, including MDD and BD, are two to three times more likely to exhibit aggression than those without mental disorders (11). The clinical characteristics of violent behavior differ significantly between BD and MDD (12). In patients with BD, the risk of violence increases significantly during manic or mixed episodes (13). In contrast, violent behavior in patients with MDD is often associated with poor regulation of negative emotions (14). Although episodes of MDD are characterized by anhedonia, low mood, low energy, and loss of interest, severe depressive states would lead to agitation, poor impulse control, and even suicidal behavior (15). Clinically, patients with MDD might exhibit self-violence, such as self-injury and suicidal behavior, particularly those with severe depressive symptoms (16, 17).

Elderly patients with a higher risk of violence experience a lower quality of life, along with increased incidence of disability and mortality (18). A meta-analysis of 35 studies found that 17% of newly admitted psychiatric patients exhibited violent behavior, indicating that mental health service providers often witness or suffer from physical injury when caring for these patients (19). Elderly individuals with mood disorders experience a decline in cognitive function as their condition progresses, leading to impaired judgment and self-control, which increases the risk of violent behavior towards healthcare providers (20). Non-pharmacological interventions play an important role in addressing the aggressive behavior of patients with mood disorders. For instance, de-escalation techniques can help patients articulate their emotions and needs through verbal and behavioral guidance, enhance their sense of participation and control, and promote emotional stability and self-esteem (21).

Although non-pharmacological interventions have shown advantages in managing violence risk, their limitations should be considered (22). Most importantly, the effectiveness of these interventions depends on the clinical conditions of individuals, as well as cultural and social factors. Identifying the specific factors contributing to violence in patients with MDD and BD can aid in developing targeted risk assessment measures and corresponding interventions. Nonetheless, relevant studies are scarce. To address this issue, the present cross-sectional study aims to explore the risk factors influencing violence levels in patients with mood disorders and to identify the differences in these factors between the clinical subtypes of bipolar disorder and major depressive disorder.

## Materials and methods

### Participants

A retrospective cross-sectional study was conducted in Guangdong, China, from February 2021 to March 2024. Participants were consecutively recruited from the geriatric psychiatric ward of a tertiary psychiatric hospital. Inclusion criteria were as follows: a) a diagnosis of BD or MDD, b) the ability to communicate and complete the scale ratings, and c) agreement to participate and complete the written informed consent. Exclusion criteria were as follows: a) the presence of severe physical diseases, and b) refusal to sign the written informed consent. The study was conducted in accordance with the Declaration of Helsinki and was approved by the Ethics Committee of the Affiliated Brain Hospital of Guangzhou Medical University.

### Measurements

All outcomes were collected within 3 days of admission, including socio-demographic characteristics, depressive symptoms, activities of daily living, and risk of violence. A specially designed questionnaire was used to gather demographic characteristics, including age, gender, education, diagnosis, smoking status, alcohol use, and other relevant factors. Methodological quality was evaluated using the checklist from the Strengthening the Reporting of Observational Studies in Epidemiology (STROBE) statement.

### Brøset Violence Checklist

The Brøset Violence Checklist (BVC), developed by Linaker et al. (1995), was used to evaluate the risk of violence among participants (23). The BVC comprises six items: confusion, irritability, boisterousness, verbal threats, physical threats, and attacking objects. Each item is scored as 0 or 1 (yes = 1; no = 0) based on the presence of risky behavior. A total score of 1–2 indicates a moderate risk of violence, while a score of 3 or higher signifies a high risk. The BVC has previously demonstrated validity and reliability in assessing violence risk (24, 25).

### The Geriatric Depression Scale

The Geriatric Depression Scale was used to evaluate depressive symptoms in older adults (26). The scale consists of 30 items, with scores ranging from 0 to 30. A score of 11 or above indicates the presence of depressive symptoms (27). Specifically, scores of 0–10 suggest no depression, 11–20 indicate mild depression, 21–25 signify moderate depression, and scores of 26 or higher indicate severe depression.

### Functional Activity Questionnaire

The Functional Activity Questionnaire, developed by Pfeffer, was used to quantify dependence in daily activities among older adults (28). The FAQ consists of 10 items that assess dependence in activities such as managing simple finances, paying bills on schedule, shopping alone for clothes, household necessities, and groceries, pursuing hobbies, doing chores, preparing meals, keeping up with current events, discussing TV programs, books, and magazines, following schedules, and maintaining the ability to be active. Each item is rated on a scale from 0 to 3, where 0 indicates no assistance needed, 1 denotes mild difficulty, 2 signifies the need for assistance, and 3 represents complete dependence on external aid. FAQ score< 5 indicates normal with the ability to independently complete social life; a score of 5 or above indicates an abnormality that individuals being dependent with the family or community.

### Data collection

Data were collected by research assistants who had completed consistency training. Before the interview, participants were informed about the purpose and significance of the study and were given instructions on how to complete the questionnaires and sign the informed consent forms. All completed questionnaires were collected on-site and checked for completeness. Out of the 141 questionnaires distributed, all 141 were included in the final analysis.

### Statistical analyses

All statistical analyses were performed using SPSS version 25.0. Continuous variables and categorical variables were presented as mean ± standard deviation (SD) or frequency (percentage), respectively. Differences in clinical and violence characteristics among participants were analyzed using t-tests and one-way ANOVA. Indicators that were statistically significant in the univariate analysis were included in the linear regression model. A *P* value < 0.05 (two-tailed) was considered statistically significant.

## Results

### Demographic and clinical variables

Among the 141 elderly patients with mood disorders, 40 (28.4%) were diagnosed with BD and 101 (71.6%) with MDD. The study included 34.8% male and 65.2% female participants, with an average age of 67.42 ± 6.91 years old. In terms of educational attainment, 12.1% had completed a bachelor’s degree or higher, 22.7% had completed senior school or an associate degree, and the majority (65.2%) had received education at the junior high school level or below. Unhealthy lifestyles were reported by a minority of participants, with smoking prevalence at 7.1% and alcohol use at 5.0%. (**Table 1**)

**Table 1:** Demographic and clinical variables.

Item	Subcategory	Frequency	Proportion (%)
Sex			
	Male	49	34.8
	Female	92	65.2
Age			
	<60	18	12.8
	60 ≤ age < 75	103	73.0
	>75	20	14.2
Education			
	Junior high school and below	92	65.2
	Senior school and associate degree	32	22.7
	Bachelor Degree and above	17	12.1
Diagnosis			
	Bipolar disorder	40	28.4
	Major depressive disorder	101	71.6
Smoking			
	No	131	92.9
	Yes	10	7.1
Alcohol use			
	No	134	95.0
	Yes	7	5.0
First admission			
	No	49	34.8
	Yes	92	65.2
Type of admission			
	Voluntary	32	22.7
	Involuntary	109	77.3
Length of hospitalization			
	≤14	23	16.3
	>14	118	83.7
Restraint			
	No	128	90.8
	Yes	13	9.2
GDS			
	Normal	38	27.0
	Abnormal	103	73.0
FAQ			
	Independent	79	56.0
	Dependent	62	44.0

### Univariate analysis of violent risk in elderly patients with mood disorders

According to the grouping characteristics of different variables, t-tests or one-way ANOVA tests were used. The results indicated that gender, unhealthy lifestyles (smoking and drinking), first admission status, type of admission, length of hospitalization, and use of restraints were associated with the occurrence of aggressive behavior in elderly patients with mood disorders (*P* < 0.05). Additionally, the GDS and FAQ scores of hospitalized elderly patients with mood disorders showed statistically significant differences (*P* < 0.05). (**Table 2**)

**Table 2:** Univariate analysis of BVC scores in patients with mood disorders.

Item		BVC	*t/F*	*P*
Sex				
	Male	1.51±1.80	25.87	0.001
	Female	0.53±1.11		
Age				
	<60	0.61±0.98	2.835	0.062
	60-74	1.04±1.60		
	≥75	0.25±0.72		
Education				
	Junior high school and below	0.72±1.33	2.225	0.112
	Senior school and associate degree	1.34±1.81		
	Bachelor Degree and above	0.82±1.33		
Diagnosis				
	Bipolar disorder	2.33±1.77	-6.974	<0.001
	Major depressive disorder	0.23±0.77		
Smoking				
	No	0.73±1.32	-3.056	<0.001
	Yes	2.70±2.00		
Alcohol use				
	No	0.82±1.42	-1.842	0.068
	Yes	1.86±1.95		
First admission				
	No	1.31±1.79	-2.395	0.019
	Yes	0.64±1.26		
Type of admission				
	Voluntary	0.19±0.59	-4.815	<0.001
	Involuntary	1.07±1.58		
Length of hospitalization				
	≤14	0.39±0.72	-2.769	0.007
	>14	0.97±1.55		
Restraint				
	No	0.70±1.32	-3.653	0.003
	Yes	2.54±1.76		
GDS				
	Normal	2.32±1.74	39.412	<0.001
	Abnormal	0.34±0.88		
FAQ				
	Independent	1.14±1.65	2.606	0.010
	Dependent	0.53±1.11		

### Univariate linear regression analysis of violent risk in elderly patients with mood disorders

Variables with statistical differences in independent sample t-tests and one-way ANOVA were set as independent variables for further analysis. Univariate linear regression analysis was performed on these variables, indicating that gender, smoking, first admission status, type of admission, length of hospitalization, GDS scores, and FAQ scores were statistically significant (**Table 3**).

**Table 3:** Univariate linear regression analysis of BVC scores in patients with mood disorders.

Item		*β*	*P*
Sex			
	Male	0.319	<0.001
	Female	-0.319	
Smoking			
	No	-0.346	<0.001
	Yes	0.346	
First admission			
	No	0.217	0.010
	Yes	-0.217	
Type of admission			
	Voluntary	0.255	0.002
	Involuntary	-0.255	
Length of hospitalization			
	≤14	-0.146	0.085
	>14	0.146	
Restraint			
	No	-0.364	<0.001
	Yes	0.364	
GDS			
	Normal	0.621	<0.001
	Abnormal	-0.601	
FAQ			
	Independent	0.207	0.014
	Dependent	-0.207	

### Linear regression analysis of violent risk in elderly patients with mood disorders

In the linear regression analysis, the BVC scores were used as the dependent variable for mood disorders overall, as well as separately for MDD and BD. The results indicated that GDS scores (abnormal), restraint condition (Yes), and length of hospitalization (>14 days) were significant factors influencing violence risk in participants with mood disorders (*P* < 0.05). For participants with MDD, restraint condition (Yes) and abnormal GDS scores were significant factors (*P* < 0.05). Meanwhile, for participants with BD, the length of hospitalization (>14 days) was a significant factor (*P* < 0.05) (**Table 4**).

**Table 4:** Multivariate linear regression analysis of BVC scores among patients with mood disorder, major depressive disorder, and bipolar disorder.

Item	Mood disorder		Major depressive disorder		Bipolar disorder	
	*β*	*P*	*β*	*P*	*β*	*P*
GDS (=Abnormal)	-0.424	<0.001	-0.207	0.015	–	–
Restraint (=Yes)	0.181	0.003	0.437	<0.001	–	–
Length of hospitalization (>14 day)	0.145	0.013	–	–	0.260	0.047

## Discussion

This cross-sectional study examined various factors influencing the risk of violent behavior in elderly inpatients with mood disorders, which aimed to explore the risk factors affecting violence levels in these patients and to identify differences in these factors between the clinical subtypes of bipolar disorder and major depressive disorder. Firstly, elderly inpatients with mood disorders generally have a high potential risk of violence. Compared to patients with dementia (29, 30), the risk of violence in those with mood disorders had been often overlooked (31). Elderly patients with BD might exhibit extreme irritability and impulsive behavior during manic episodes, while those with MDD may become aggressive due to severe depression. Previous research has found that individuals experiencing manic episodes have a 2.6 times higher risk of violence compared to those not in a manic episode (31). Notably, our findings indicated that elderly patients with BD had a higher risk of violence compared to those with MDD (BVC: BD *vs*. MDD, *P* < 0.001). Therefore, it is crucial to conduct thorough violence risk assessments for elderly inpatients with mood disorders and to implement early interventions for high-risk individuals. Moreover, enhancing the capacity of healthcare providers to manage aggressive patients is also essential.

Secondly, in elderly patients with MDD, we observed that individuals with higher BVC scores also had higher GDS scores, suggesting that aggressive behavior was associated with the severity of depressive symptoms. This finding is consistent with a previous study by Corruble (1999) (32). However, a recent review by Weltens et al. (2021) found no clinical association between violence risk levels and the severity of depressive symptoms in patients with MDD (33). The discrepancy between results may be attributed to differences in study populations, given that Weltens et al. (2021) focused on adult patients, while our investigation specifically examined elderly patients with MDD.

Thirdly, the BVC score was associated with the use of physical restraint among patients with mood disorders and MDD. However, this result should be interpreted with caution. In general, national guidelines stipulate that physical restraint is implemented when a patient exhibits violent behavior towards others, self-injury (or suicide), or disturbances in medical order (34). The items on the BVC encompass aggressive behaviors, and most of these items are also regarded as indicators of physical assault according to clinical guidelines (35). Besides, empirical studies have shown that aggressive behavior is a key predictor of physical restraint, with 70% of patients with MDD potentially posing physical harm to themselves or others (36, 37). Therefore, significant attention should be given to the violence risk in patients with MDD, while the BVC may serve as a concise and convenient tool for assessing this risk.

Last but not least, among patients with BD, those with higher BVC scores at admission had longer hospitalizations. On the one hand, a high BVC score often reflects the severity of a manic episode, necessitating an extended hospitalization (typically >14 days) due to the increased need for mental health care (38, 39). On the other hand, prolonged hospitalization might exacerbate feelings of anxiety and tension among elderly patients with BD, potentially increasing the risk of violence (8). Mania, closely related to violence risk, is a core symptom of BD (40). However, managing BD during hospitalization is challenging due to the alternating episodes of mania and depression. Thus, continuous evaluation of violence risk in elderly patients with BD is essential throughout during inpatient treatment.

In conclusion, patients with mood disorders have a potential risk of violence, with clinical factors differing between BD and MDD. Thus, concise risk assessments and early interventions are essential to manage and mitigate violence risk. Nevertheless, this study has several limitations that should be noted. First, BVC was selected for its strength in rapid violence risk assessment, but the multifaceted nature or long-term factors of violence risk should be also taken into account, and the assessment was unable to quantify the severity of manic/mixed episodes. Second, crucial clinical history characteristics, such as the number of previous episodes and suicide attempts, were not collected, limiting the analysis of these important prognostic factors. Third, the retrospective cross-sectional design inherently prevents the establishment of causal inferences regarding the directionality of the observed associations. Future research should address these limitations to provide a more comprehensive understanding of violence risk in patients with mood disorders.

## Data Availability

The raw data supporting the conclusions of this article will be made available by the authors, without undue reservation.
